# Evaluation of an e-Learning Program for Community Pharmacists for Dispensing Emicizumab (Hemlibra) in France: Nationwide Cross-Sectional Study

**DOI:** 10.2196/54656

**Published:** 2024-04-04

**Authors:** Valérie Chamouard, Julie Freyssenge, Béatrice Clairaz-Mahiou, Felicia Ferrera Bibas, Laurie Fraticelli

**Affiliations:** 1 Louis Pradel Hospital, Groupement Hospitalier Est, Hospices Civils de Lyon French Reference Center of Hemophilia Bron France; 2 University Claude Bernard Lyon 1 INSERM U1290 Research on Healthcare Performance RESHAPE Lyon France; 3 French Society of Pharmaceutical Sciences Châtenay-Malabry France; 4 Provence-Alpes-Cote-d’Azur Unions for health professionals Marseille France; 5 Laboratory P2S (Health Systemic Process), UR 4129 University Claude Bernard Lyon 1 Lyon France

**Keywords:** hemophilia, care pathway, emicizumab, Kirkpatrick model, pharmacy, survey, Hemlibra, France, e-learning program, pharmacists, pharmacist, hemophilia A, hospital, HEMOPHAR, methodology, community, engagement, pharmaceutical, rare disease, digital health, intervention

## Abstract

**Background:**

Since June 2021, patients with hemophilia A with antifactor VIII inhibitors and those with severe hemophilia A without antifactor VIII inhibitors treated with Hemlibra have had to choose between a community or hospital pharmacy. The French reference center for hemophilia developed the HEMOPHAR e-learning program for community pharmacists for dispensing emicizumab.

**Objective:**

This study aims to evaluate the efficiency and safety of this new care pathway by assessing the HEMOPHAR e-learning program.

**Methods:**

The methodology is based on Kirkpatrick’s model for evaluating the immediate reaction of trained community pharmacists (level 1), their level of acquired knowledge (level 2), and their professional practice after 3 months of dispensation (level 3).

**Results:**

The HEMOPHAR e-learning program reached a large audience, with 67% (337/502) of the eligible community pharmacists following it. The immediate reaction was overall satisfying. High rates of engagement were reported with 63.5% (214/337) to 73.3% (247/337) of completed training modules, along with high rates of success with quizzes of 61.5% (174/337) to 95.7% (244/337). We observed that 83.9% (193/230) of the community pharmacists needed less than 2 attempts to pass the quiz of the module related to professional practice, while the other quizzes required more attempts. Advice on compliance and drug interactions were most frequently provided to patients by the community pharmacists.

**Conclusions:**

This study suggests ways to improve the training of community pharmacists and to optimize coordination with treatment centers. This study also reports on the feasibility of switching to a community pharmacy in a secure pharmaceutical circuit, including in the context of a rare bleeding disease.

**Trial Registration:**

ClinicalTrials.gov NCT05449197; https://clinicaltrials.gov/study/NCT05449197

**International Registered Report Identifier (IRRID):**

RR2-10.2196/43091

## Introduction

While community pharmacists have long served as safety consultants for patients with minor illnesses, the literature highlights the multifaceted and evolving role of the community pharmacists to improve care pathways especially for patients requiring urgent care [1] or for the management of chronic diseases [2-4]. Evidence of the effectiveness of community pharmacists’ interventions exists for the management of chronic diseases [4], vaccination [5], and optimization of transitions of care [6,7]—the latter referring to the movement of patients across institutions, among providers, between different levels of care, and to and from home. The main pitfall that may occur during these transitions is discontinuity in the patient’s care pathway, leading to potential complications [8]. But offering standardized training regarding processes and procedures for all the involved stakeholders has shown to be a facilitator [9].

The transition of care has typically been implemented in France for patients with hemophilia A with or without inhibitors. This disease is a rare bleeding disorder whose treatment relies on factor VIII injections or bypass agents. Chronic treatment for hemophilia A has been available only in French hospital pharmacies, and monthly dispensing was conditional on reimbursement of these drugs [10]. In France, the delivery of drugs to patients’ homes is outstanding and is only legally conceivable in specific cases [11]. Access to treatment could, in some cases, be a challenge as the burden of the disease can be significant [12]. Hence, a new organizational scheme has been proposed for patients treated with emicizumab (Hemlibra) [13] in France.

Since June 15, 2021, community pharmacists have played an important role in the care pathway with the implementation of a dual circuit of dispensing, providing the patient the freedom to choose the site from which to pick up emicizumab: hospital or community pharmacy. For now, this dual circuit is only possible for emicizumab for routine prophylaxis to prevent or reduce the frequency of bleeding episodes in adults and children of all ages—newborn and older. Treatment is initiated at French hospital-based reference centers for hemophilia with the injection of loading doses during consultations where the patient also benefits from treatment education sessions. While patients’ satisfaction with the product has already been evaluated [14], the dual circuit of dispensing of emicizumab remains to be evaluated.

The French reference center for hemophilia has chosen to develop the HEMOPHAR e-learning program and propose this training program to community pharmacists to dispense emicizumab, as soon as initial maintenance doses are established, to preserve the quality and safety of the patient-facing service. Although the training of community pharmacists is not mandatory by the French health authorities, the hemophilia treatment centers strongly encourage community pharmacists to follow the HEMOPHAR e-learning program by systematically sending an email invitation with personalized log-in details, as soon as the patient has chosen the community pharmacy.

More than 1 year after the implementation of this training program, a nationwide study called “PASODOBLEDEMI I” was conducted to evaluate the direct reaction of the community pharmacists following the implementation of the HEMOPHAR e-learning program and the use and functionalities of the available training resource, and to evaluate their professional practice at least 3 months after the first dispensation of emicizumab.

## Methods

### Content of the HEMOPHAR e-Learning Program

Community pharmacists receive personal log-in codes for the HEMOPHAR e-learning program from the national reference center for hemophilia (Lyon, France). They can take the course at any time on working days. They can also interrupt the program and resume it at a later date without losing their progress through the program. The program comprises four e-learning modules: (1) presentation of the disease, (2) therapeutic management, (3) organization of care, and (4) practice in the community pharmacy (Multimedia Appendix 1). At the end of each module, the community pharmacist can participate in a quiz composed of 4 or 5 questions. Quizzes are mandatory to obtain the certificate of achievement (with at least 80% of correct answers), but this certificate is not mandatory to dispense emicizumab.

### Study Design

We designed a cross-sectional study based on the Kirkpatrick model [15] based on 2 closed surveys and on an analysis of secondary data. The evaluation of the training program comprised the investigation of 3 information levels.

Level 1, related to “Reaction,” referred to the evaluation of the immediate feedback of community pharmacists who attended the HEMOPHAR e-learning program. Invitations to complete e-questionnaire were sent via email to community pharmacists who completed the training and obtained the certificate of achievement within 48 hours post training. The e-questionnaire was intentionally short and composed of 8 questions, including 5 questions based on a 4-item Likert scale (“not at all satisfied,” “not very satisfied,” “somewhat satisfied,” and “very satisfied”) and 3 closed-ended questions (yes/no). The estimated time to complete this e-questionnaire is approximately 1 minute. This questionnaire is informative to help determine which elements of the training program are effective or appreciated, and which ones need to be improved.

Level 2, related to “Learning,” referred to knowledge acquisition during the program. This level consists of accurately measuring the knowledge acquired during the active training modules. In contrast to immediate reactions to training (level 1), this second level allows for the measurement of specific outcomes by collecting data regarding usage targets such as participation rate, completion rate, or time spent on training to supplement participant feedback.

Level 3, related to “Behavior,” consisted of evaluating the professional practice of community pharmacists at least 3 months after the first dispensation of emicizumab, to determine whether they have followed the HEMOPHAR e-learning program. Specific e-questionnaires were elaborated using Likert-based scoring for both trained and untrained community pharmacists dispensing emicizumab. Level 3 consists of determining whether the training program has a direct impact on the participants’ dispensing behaviors. The community pharmacists were informed about the purpose of and estimated time required to complete the survey via email before opting to complete the e-questionnaire. The level 3 e-questionnaire was composed of 13 multiple-choice questions. The estimated time to complete this questionnaire is approximately 8 minutes.

The contents of the questionnaires were initially developed by a multidisciplinary team composed of hospital and community pharmacists and research scientists specialized in methodology and care accessibility. A scientific committee was specifically constituted for the PASODOBLEDEMI I study to challenge and validate the purposes, methodology, questionnaires, and expected outcomes. This committee was coordinated by the national reference center for hemophilia and composed of hospital and community pharmacists, physicians, and the French association of patients with hemophilia (French: Association Française des Hémophiles). Following the recommendations of the scientific committee, the content of the e-questionnaires has evolved by integrating operational and organizational aspects and optimizing literacy. The e-questionnaires were finally tested in real-life conditions on a sample of eligible populations. The content of the questionnaires was finally validated by the scientific committee in July 2022.

### Sample Size

**Figure 1:**
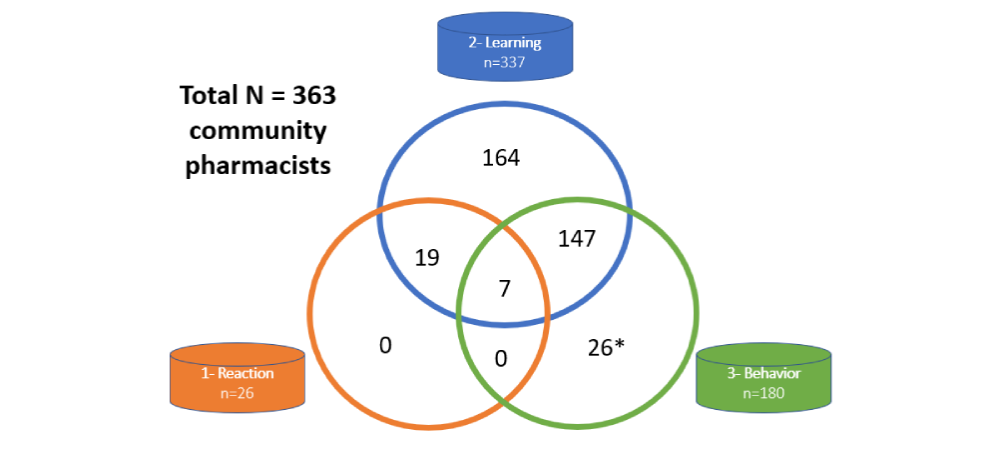
Number of community pharmacists included in the 3 levels of the PASODOBLEDEMI I study. *A total of 26 community pharmacists have either completed the Roche training (n=8) or have not completed any training (n=18).

Since emicizumab became available in French community pharmacies, from June 2021 to December 2022, a total of 337 volunteer community pharmacists followed at least 1 of the 4 training modules available in the HEMOPHAR e-learning program. The expected minimum sample size for the questionnaire related to the reaction of the community pharmacists (level 1) was intended to encompass the maximum number of the trained participants who obtained the certificate of achievement during the month preceding the study start date. From July to December 2022, in total, 26 of the 337 community pharmacists completed the short e-questionnaire immediately after following the HEMOPHAR e-learning program, representing the Reaction sample (level 1). As the answers were not divergent, we did not consider it appropriate to extend the duration of data collection. At least 3 months after the implementation of the dual circuit of dispensing, 180 community pharmacists completed the e-questionnaire concerning their professional practice. The Behavior sample (level 3) contains community pharmacists who followed at least 1 of the 4 modules in the HEMOPHAR e-learning program (154/337, 45.7%). The expected minimum sample size for this questionnaire was at least 30%, which has been far exceeded. A total of 363 community pharmacists were included in this study, representing 67% of the eligible community pharmacists in France (337/502 active community pharmacies, last updated in September 2022; Figure 1).

### Statistical Analysis

Baseline characteristics were described by counts and percentages for categorical variables and medians and IQRs for continuous variables, mentioned in parentheses in the following order in the Results section: community and hospital pharmacy. Statistical analysis was performed using R (version 4.1.1; The R Foundation) software.

### Ethical Considerations

The study complies with reference methodology MR-004 (“related to research not involving the human person, studies and evaluations in the health field”) [16]. This French legislation mentions that the persons involved in the research or their legal representatives (or both) are informed beforehand and individually of any processing of their personal data for the purposes of research for the present methodology (Title II 2.5.1 related to information of participants); therefore, it did not require written consent from the study participants. No incentive was offered to participate in the survey. All the collected data from community pharmacists were nonsensitive and were stored by the study sponsor (Hospices Civils de Lyon) on a secure server for 15 years. The ethics committee of the Hospices Civils de Lyon approved the study (n°2022-06-01, obtained on June 14, 2022). The PASODOBLEDEMI I study was registered in ClinicalTrials.gov (NCT05449197; related to the evaluation of Reaction, Learning, and Behavior)*.*

## Results

### Level 1: Immediate Reaction After the HEMOPHAR e-Learning Program

Immediate satisfaction with the HEMOPHAR e-learning program was high, with 18 (69.2%) community pharmacists very satisfied and 8 (30.8%) somewhat satisfied (Table 1). Nearly 3 out of 4 community pharmacists were very satisfied because the training content met their expectations (n=17, 67.4%) and was considered relevant to their professional practice (n=19, 73.1%). They were less very satisfied with the communication format (n=14, 53.8%) and the duration of the training (n=13, 50.0%). Overall, 8 (30.8%) community pharmacists planned to follow another learning program. Finally, 96.2% (25/26) of them would recommend the HEMOPHAR e-learning program to their colleagues.

**Table 1:** Satisfaction levels recorded immediately after the HEMOPHAR e-learning program (N=26).

Satisfaction levels		Participants, n (%)
**Overall level of satisfaction**		
	Very satisfied	18 (69.2)
	Somewhat satisfied	8 (30.8)
	Not very satisfied	0 (0)
	Not at all satisfied	0 (0)
**The training content meets expectations**		
	Very satisfied	17 (67.4)
	Somewhat satisfied	8 (30.8)
	Not very satisfied	0 (0)
	Not at all satisfied	1 (3.8)
**Satisfaction with the communication format**		
	Very satisfied	14 (53.8)
	Somewhat satisfied	11 (42.3)
	Not very satisfied	1 (3.8)
	Not at all satisfied	0 (0)
**The training is relevant to professional practice**		
	Very satisfied	19 (73.1)
	Somewhat satisfied	6 (23.1)
	Not very satisfied	0 (0)
	Not at all satisfied	1 (3.8)
**Satisfaction with the duration of training**		
	Very satisfied	13 (50.0)
	Somewhat satisfied	10 (38.5)
	Not very satisfied	2 (7.7)
	Not at all satisfied	1 (3.8)

### Level 2: Learning and Knowledge Acquired During the Training

A median delay of approximately 9 (IQR 3-21) days was observed between the day the community pharmacists received their connection codes and the day they logged in to the HEMOPHAR e-learning program. Among the community pharmacists who started at least 1 of the 4 training modules, 81.9% (276/337) of them started all the training modules available in the HEMOPHAR e-learning program, and 40.9% (113/276) of them completed it. The training modules related to the presentation of the disease and the one related to therapeutic management reached most of the community pharmacists, with more than 80% of them having started the training modules (Table 2). Once the training modules were started, between 60% and 75% of community pharmacists completed them.

**Table 2:** Statistics for the training follow-up modules available to community pharmacists in the HEMOPHAR e-learning program.

Training modules	Cumulated duration of the videos	Started (n=337), n (%)	Completed^a^ (relative to those who started^b^), n (%)	Successful quiz completers (relative to those who started^b^), n (%)
1. Presentation of the disease	16 minutes 52 seconds	280 (83.1)	214 (63.5)	214 (76.4)
2. Therapeutic management	22 minutes 51 seconds	283 (84.0)	247 (73.3)	174 (61.5)
3. Organization of care	22 minutes 25 seconds	255 (75.7)	244 (72.4)	244 (95.7)
4. Practice in the community pharmacy	15 minutes 46 seconds	230 (68.2)	223 (66.2)	183 (79.6)

^a^Community pharmacists who completed the training modules, with or without attempting the related quizzes.

^b^Denominators are the number of community pharmacists who started the training module (n=337).

**Figure 2:**
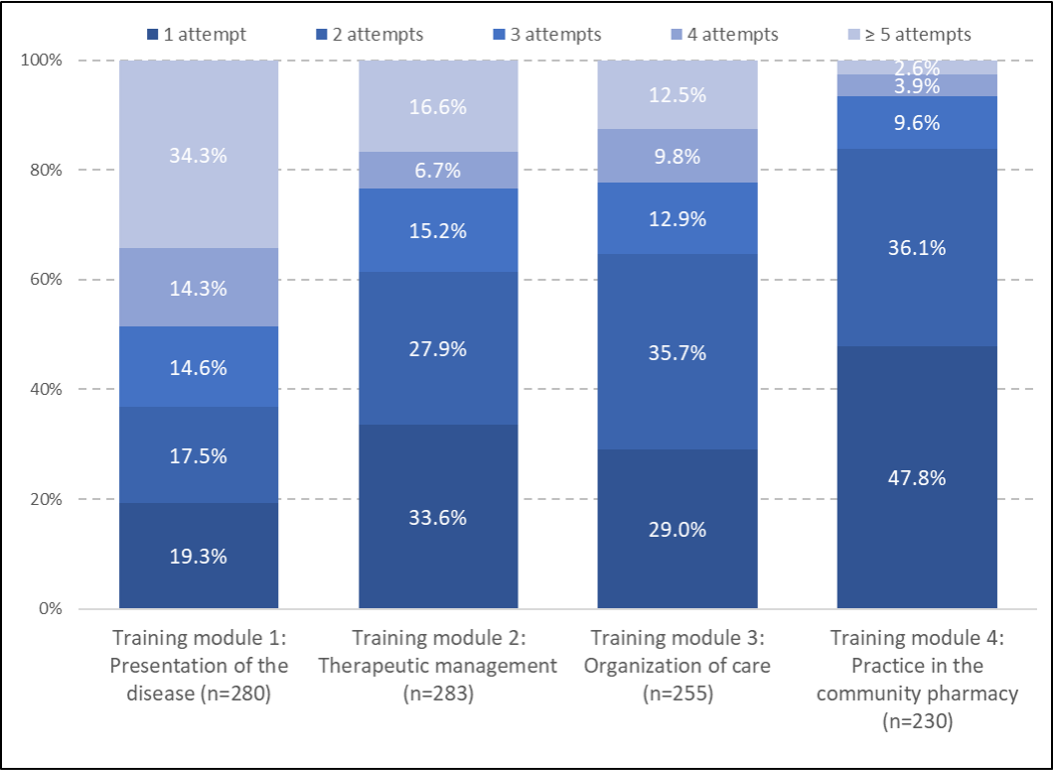
Number of attempts at the quizzes for each of the training modules in the HEMOPHAR e-learning program.

Community pharmacists usually need more than 1 attempt to pass the quiz at the end of each module (Figure 2). The quiz related to the organization of care was the most successful with a 95.7% success rate associated with the lowest number of attempts. The time spent per quiz varied greatly with 50% of the community pharmacists, who needed 6.8 minutes for the presentation of the disease module, 3.5 minutes for the therapeutic management module, 1.5 minutes for the organization of care module, and 2.6 minutes for practice in the community pharmacy module.

### Level 3: Behavior During Professional Practice of Community Pharmacists

The community pharmacists reported that mostly 1 patient chose their pharmacy for dispensing emicizumab (90.9%, 140/154 unique community pharmacies). Nearly half of the community pharmacists (74/154, 48.1%) who responded to the Behavior questionnaire followed the HEMOPHAR e-learning program for more than 3 months, and one-third of the sample (51/154, 33.1%) followed the program for more than 12 months.

**Figure 3:**
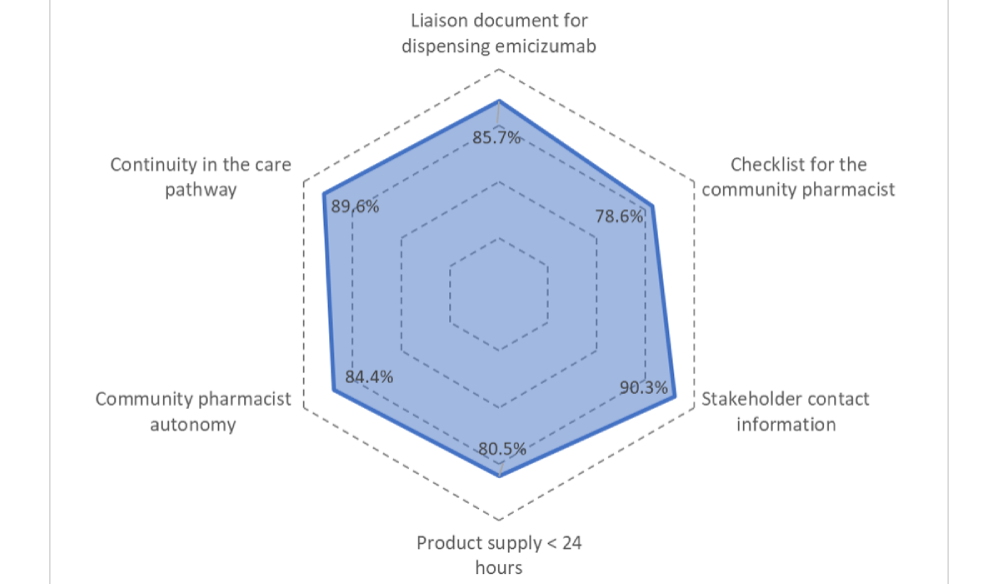
Organizational items reported from the community pharmacists who followed the HEMOPHAR e-learning program (n=154).

During their professional practice, community pharmacists may be faced with organizational constraints. Even though the HEMOPHAR e-learning program covers all aspects of organization and documentation management, community pharmacists have reported that they did not always rely on the documents available (Figure 3). For example, the most used document was the one containing the stakeholders’ contact information (90.3%, 139/156) and the liaison document for emicizumab (85.7%, 132/154), unlike the checklist, which was used for only 78.6% (121/154) of the community pharmacists. Overall, 89.6% (138/154) of community pharmacists reported that they did not observe any discontinuity in the pathway of care of the patient in general. Those who encountered problems of continuity of care reported constraints in terms of personnel, particularly during the holiday season, and those relating to the supply of emicizumab or those due to a prescription delay by the hospital, even though 76% (117/154) of respondents reported that patients systematically anticipate the renewal of prescription before their arrival at the community pharmacy. About 84.4% (130/154) of the community pharmacists reported that they did not need a third party to manage the dispensation until now (autonomy). Regarding the time spent in supplying emicizumab, 80.5% (124/154) of the community pharmacists received the drug in less than 24 hours.

**Figure 4:**
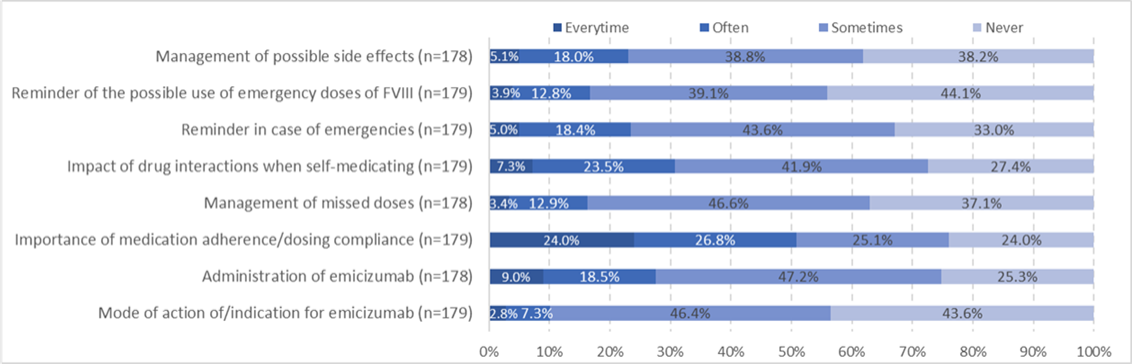
Distribution of the frequency of community pharmacists’ advice provided to patients or their caregivers, relatives, or legal representatives when dispensing emicizumab (n=154). FVIII: factor VIII.

Community pharmacists who followed the HEMOPHAR e-learning program have reported the frequency of advice provided when dispensing emicizumab (Figure 4). The most frequent advice provided by the community pharmacists concerned the importance of medication adherence, with 25.3% (36/154) of the trained community pharmacists having provided this advice time systematically at every visit of the patient or their caregiver and 24.7% (38/154) having provided this advice often. Administration of emicizumab was addressed sometimes, according to 48.1% (74/154) of the community pharmacists, as well as the impact of drug interactions when self-medicating (41.6%, 64/154; sometimes). The mode of action of emicizumab was the least discussed topic between patients and community pharmacists. Most pharmacists felt they had sufficient information for dispensing emicizumab and managing their relationship with patients (92.2%, 142/154). However, we observed that 36.4% (56/154) of community pharmacists trained with the HEMOPHAR e-learning program expressed the need for therapeutic education support.

## Discussion

The PASODOBLEDEMI I study represents the first nationwide survey to evaluate the systemic impact of the implementation of the dual dispensing circuit, quickly after its implementation, with high institutional expectations. The strengths of the PASODOBLEDEMI I study lie in its holistic approach using the Kirkpatrick evaluation model [15] and the high-quality representative sample, which enhances the validity of the findings, especially for the Behavior sample in evaluating professional practice at 3 months. We report 36% (180/502) representativeness, with more than 30% representation from each French department, reaching 65% in Hauts-de-France and 63% in Auvergne Rhône-Alpes. Representativeness was lesser for Provence-Alpes-Côte d’Azur (18%) and Ile-de-France (21%) but remained consistent with that reported in other surveys [17,18]. The size of the Reaction sample was considered sufficient (n=26) because of the convergence of the satisfaction ratings.

The HEMOPHAR e-learning program reached a large audience with 67% of the community pharmacists being eligible. The immediate reaction was overall satisfying despite mixed feedback related to the format and duration, which will be reviewed for the next version of HEMOPHAR (ie, version 2). High rates of engagement were reported, with 63.5% to 73.3% of training modules having been completed and high rates of success of 61.5% to 95.7% with the quizzes. As it is possible to take the quiz without attending the training modules, we hypothesized that some community pharmacists tested their knowledge to auto-evaluate their need for training. As an example, 76.4% of them succeeded in the quiz related to the presentation of the disease, while 63.5% of them completed the related training module. We also observed that 83.9% of the community pharmacists needed less than 2 attempts to pass the quiz in the module related to professional practice, while the other quizzes required more attempts. Advice on compliance and drug interactions were most frequently provided to patients by the community pharmacists. The mode of action of emicizumab was less frequently mentioned, which may reflect the effectiveness of the hospital education that patients received.

The e-learning format was evaluated as the best way to educate community pharmacists for dispensing emicizumab and helping them to play an important role with this patient community in primary care [19,20] because the e-learning program provides both theoretical knowledge and practical skills for health professionals and educational reinforcement for patients [21]. Comparison with other training methods, such as classroom teaching or lectures, has shown that e-learning is associated with equally satisfactory results [22]. The HEMOPHAR e-learning program represents a strong model for effective training and skill development among community pharmacists, by offering accessibility, flexibility, cost-effectiveness, multimodal learning, tracking and assessment, and updateability. Moreover, community pharmacists can return to the training modules at any time to review the videos or to download useful documents. A complementary approach would comprise affective peer coaching to increase the uptake and effectiveness of a variety of community pharmacist-led enhanced patient care services [23]. Also, the gamification of training programs presents a promising perspective for the advancement of training and development efforts by offering several advantages, including increased learner engagement, immediate feedback, collaborative learning, development of critical thinking skills, and data-driven optimization [24].

The main limitation of the PASODOBLEDEMI I study is the absence of a control group (ie, untrained community pharmacists). We were constrained by regulatory issues from accessing the list of community pharmacies chosen by the patients from among hemophilia treatment centers. So, we first relied on the community pharmacists who attended the HEMOPHAR e-learning program and then publicly invited all the eligible community pharmacists through social networking platforms (LinkedIn, Facebook, Twitter, etc) and academic societies (French society of pharmaceutical sciences, unions for health professionals, etc) to take the survey.

In France, the short training by telephone on the proper use of the product provided by Roche-Chugai to community pharmacists and validated by the French authorities was not sufficient to optimize the dual circuit for dispensing emicizumab. The HEMOPHAR e-learning program is much broader and goes beyond the strict use and storage of the product to increase the role of community pharmacists in the management of this rare bleeding disorder, becoming a new model of care [25]. Other countries have made the same decision in evolving the dispensing circuit such as Australia [26]. If hemophilia treatment centers ensure adequate education and training on the technique for subcutaneous injection for patients, community pharmacists are not, to our knowledge, trained to dispense emicizumab. Other initiatives have been developed for enhancing the role of community pharmacists for consultation services [1] and for urgent care [27], which could result in increased confidence and capability in offering the service, with many community pharmacists recognizing and embracing a shift in their roles. An evaluation of patients’ and carers’ satisfaction will be the next step in the PASODOBLEDEMI II study (registered in ClinicalTrials.gov NCT05450640).

The PASODOBLEDEMI I study provides a holistic approach based on the Kirkpatrick model for evaluating the reaction, learnings, and behaviors of community pharmacists after attending the HEMOPHAR e-learning program. With high engagement rates, the community pharmacists benefited from high quality and relevance of the content on autonomy, flexibility, and interactivity, and encompassing the whole national territory. A future certification program will provide recognition for community pharmacists’ educational efforts. The success of the HEMOPHAR e-learning program in the context of the emicizumab dual dispensing circuit may pave the way for other treatments to be made available in community pharmacies, reaching institutional expectations and enhancing the access to treatment for patients with rare diseases.

## Appendix

Multimedia Appendix 1 Screenshots of the e-learning program HEMOPHAR.

## Data Availability

The data that support the findings of this study are available from the corresponding author upon request.
