# Supplementary material for: Evaluation of an e-Learning Program for Community Pharmacists for Dispensing Emicizumab (Hemlibra) in France: Nationwide Cross-Sectional Study
Source: JMIR Form Res. 2024 Apr 4;8:e54656. doi: 10.2196/54656 (PMC11027057; doi:10.2196/54656)

Supplementary material

Module 1: Presentation of the disease

Quizz of the module 1: Presentation of the disease


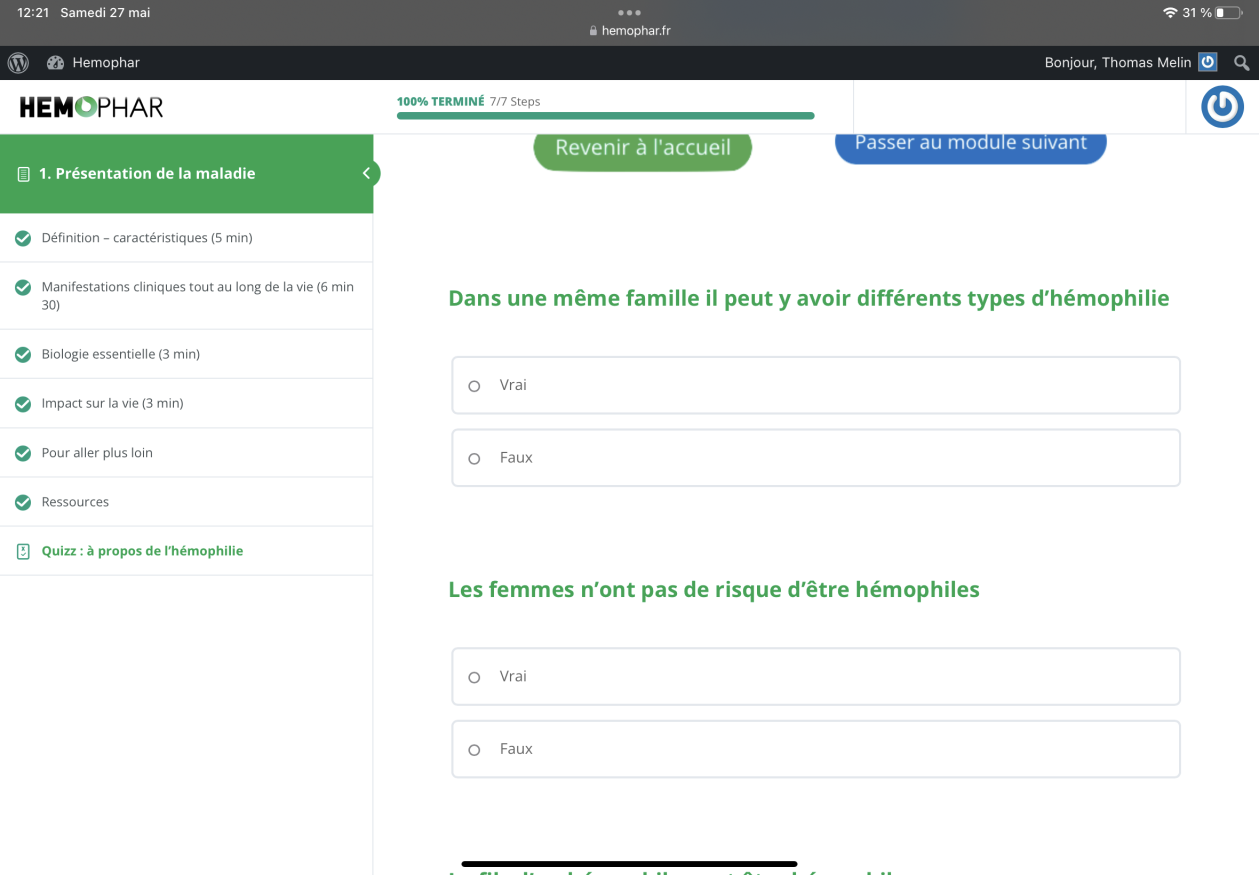


Module 2: Therapeutic management

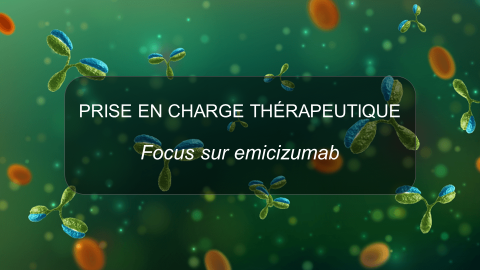


Quizz of the module 2: Therapeutic management


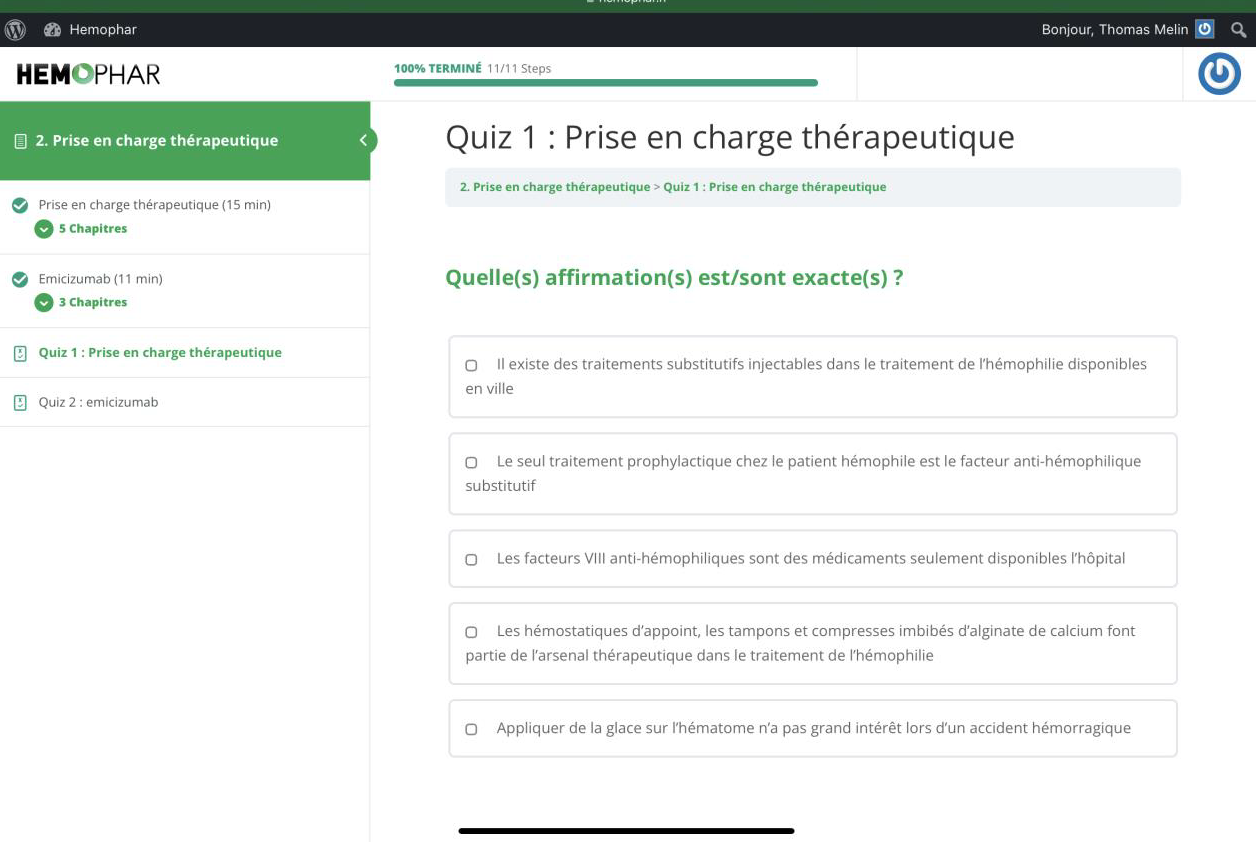


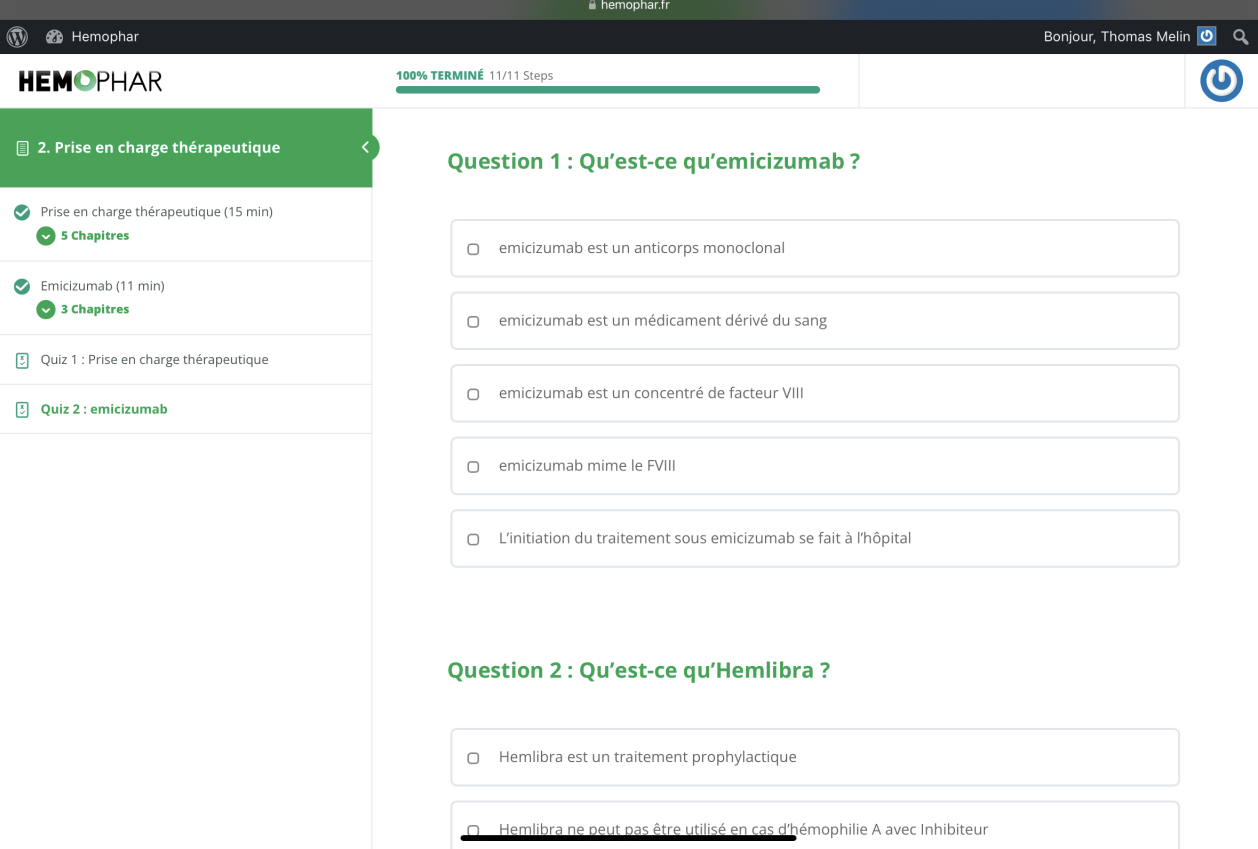


Module 3: Organization of care

Quizz of the module 3: Organization of care

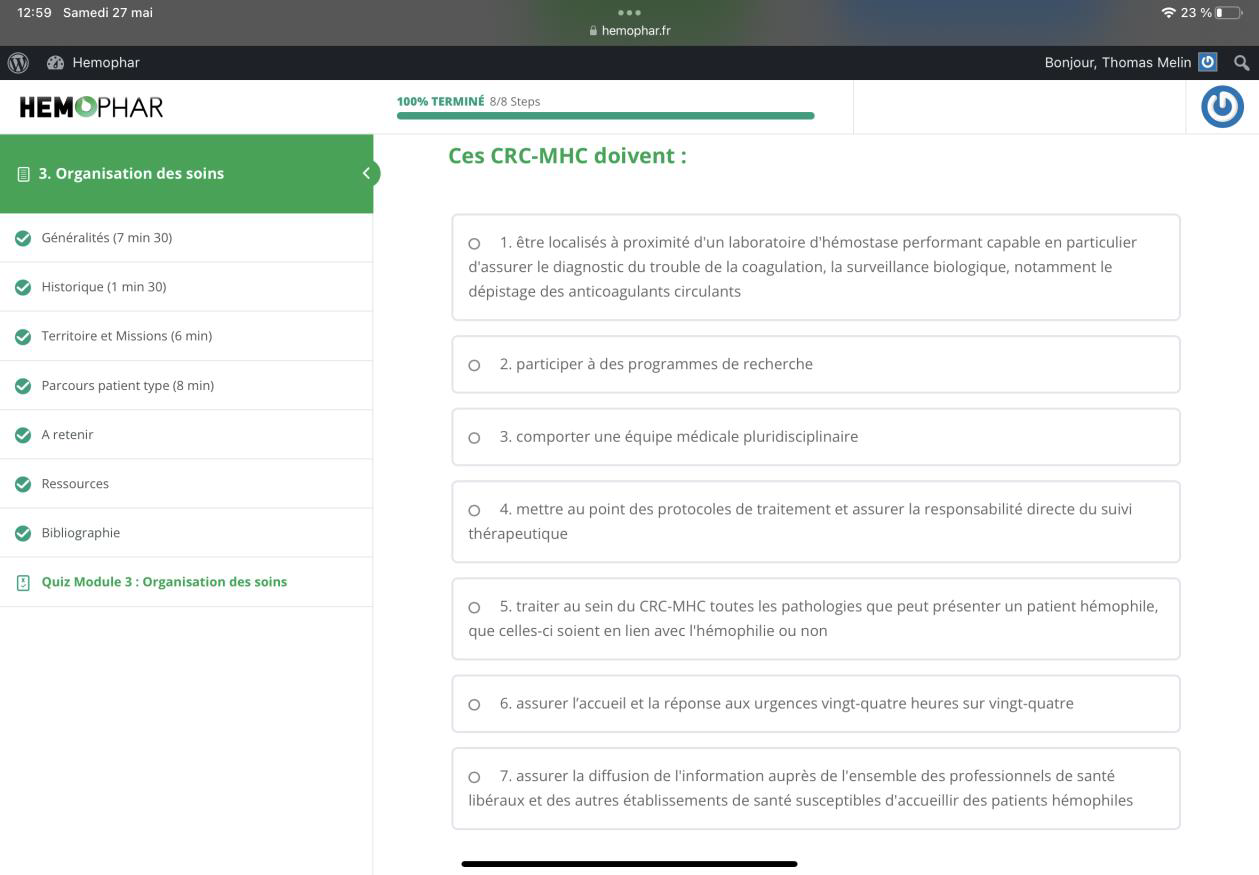


Module 4: Practice in the community pharmacy

Quizz of the module 4: Practice in the community pharmacy

Specimen of certificate of success


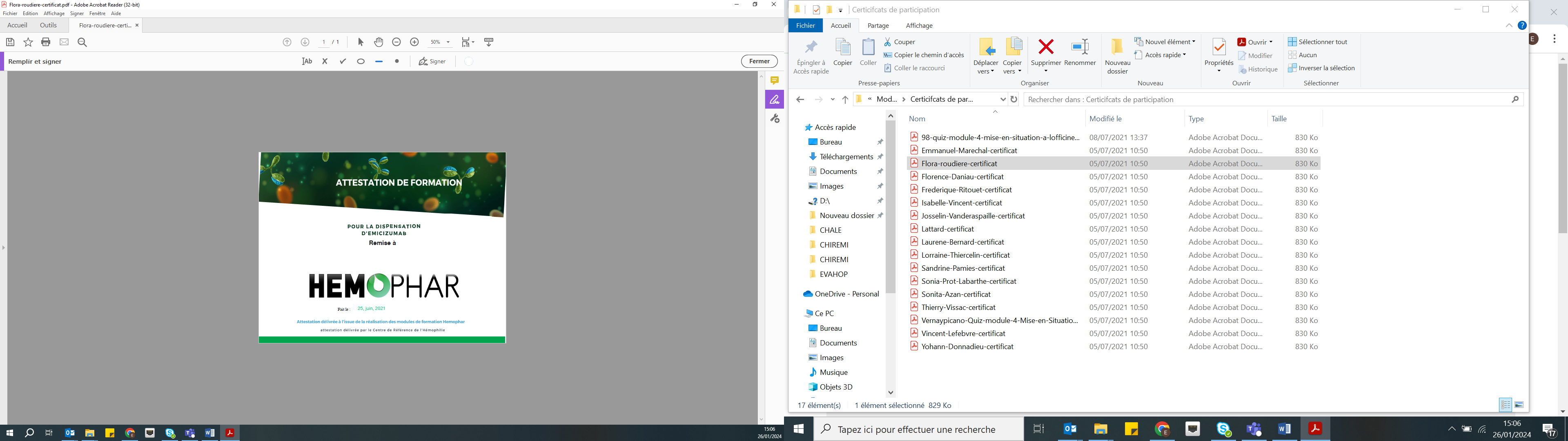

Supplement: Multimedia Appendix 1 [file formative_v8i1e54656_app1.docx]
